# Clinical applications of contactless photoplethysmography for monitoring in adults: A systematic review and meta-analysis

**DOI:** 10.1017/cts.2023.547

**Published:** 2023-05-15

**Authors:** Melissa Joanne Bautista, Mikolaj Kowal, Daniel G. W. Cave, Candice Downey, David G. Jayne

**Affiliations:** University of Leeds, Leeds, UK

**Keywords:** Monitoring, meta-analysis, systematic review, vital signs, heart rate, photoplethysmography, contactless monitoring, camera

## Abstract

Contactless photoplethysmography (cPPG) is a method of physiological monitoring. It differs from conventional monitoring methods (e.g., saturation probe) by ensuring no contact with the subject by use of a camera. The majority of research on cPPG is conducted in a laboratory setting or in healthy populations. This review aims to evaluate the current literature on monitoring using cPPG in adults within a clinical setting. Adhering to the Preferred Items for Systematic Reviews and Meta-analysis (PRISMA, 2020) guidelines, OVID, Webofscience, Cochrane library, and clinicaltrials.org were systematically searched by two researchers. Research articles using cPPG for monitoring purposes in adults within a clinical setting were selected. Twelve studies with a total of 654 individuals were included. Heart rate (HR) was the most investigated vital sign (*n* = 8) followed by respiratory rate ((*n* = 2), Sp02 (*n* = 2), and HR variability (*n* = 2). Four studies were included in a meta-analysis of HR compared to ECG data which demonstrated a mean bias of –0.13 (95% CI, –1.22–0.96). This study demonstrates cPPG can be a useful tool in the remote monitoring of patients and has demonstrated accuracy for HR. However, further research is needed into the clinical applications of this method.

## Background

Photoplethysmography (PPG) is an optical technique used to detect pulsatile blood volume changes on the skin’s surface. These blood volume changes provide information on physiological parameters such as heart rate (HR) and oxygen saturation levels (Sp02). Given the noninvasive, cheap, and accurate qualities of PPG, it is currently used in a number of medical and commercial devices [1]. One example of a PPG device is a pulse oximeter, where a probe is applied to the fingertip or earlobe to provide information on HR and Sp02.

Contactless PPG (cPPG) is a relatively novel adaptation of PPG which uses a camera to detect the pulsatile blood volume changes in the blood vessels of a person’s face. The technology was developed based on the limitations of conventional PPG. These include the need to be in contact with the skin and the measurements being restricted to the local blood flow over a few millimeters [2]. The cPPG technique is a noncontact method of measuring physiological parameters [3]. During times when remote consultations and physical distancing is beneficial, this technology can provide a diagnostic benefit in a number of clinical settings. CPPG by be beneficial in a number of clinical settings, especially when monitoring causes distress to patients, for example in those with delirium, head injuries, or children. This technology is also ideally suited for remote monitoring especially with the use of web cameras and smartphone technology.

Since cPPG was first described, the focus of research has been on demonstrating the technology’s feasibility and application in controlled laboratory settings [4]. Information on how this technology functions in clinical settings is limited.

### Primary Objective and Secondary Objectives

This systematic review aims to summarize the current literature on the clinical applications of cPPG monitoring in adults. Clinical applications were defined as either set in a clinical environment or the assessment of vital signs in patients with a particular clinical condition.

The secondary objectives include collating available data on accuracy and the limitations of cPPG when used in clinical settings.

## Methods

The search terms are available in supplementary material and this review adheres to the Preferred Reporting Items for Systematic reviews and Meta-analyses (PRISMA) guidelines [5].

### Study Selection Criteria

*Population*: This study was limited to adults.

*Intervention:* Contactless vital signs monitoring using cPPG methods.

*Environments*: Given the nature of the research question, only studies conducted within a clinical setting or where the aim was to assess a clinical question were included.

*Comparator:* All studies that met the inclusion criteria, regardless of whether or not a comparator was described, were included. Where a comparator was described, further analysis was conducted.

*Outcomes:* Outcomes of interest included the detection rate and accuracy of any physiological parameters (HR, respiratory rate (RR), saturation level, and blood pressure). Where available, further details regarding the acceptability, adverse events, and limitations were included.

*Exclusion criteria*: Non-English studies, review articles, and conference abstracts were excluded.

### Search Strategy and Study Selection

An initial limited search was conducted to identify the appropriate search terms and available synonyms. The initial search terms were PPG and camera. The final search terms can be found in supplementary material.

OVID (Medline, Embase), Web Of Science, Cochrane Library, and clinicaltrials.org were systematically searched to include abstracts, ongoing trials, and protocols.

The search was applied from database inception to February 2022. All abstracts were screened against the predetermined inclusion and exclusion criteria. The relevant abstracts were then reviewed to determine relevance. References of relevant articles were screened for possible eligible studies. The final research articles were then screened against the protocol by a second reviewer (MK).

### Data Extraction

Data extraction was completed by two independent researchers (MB and MK) using a pre-determined data extraction tool on Microsoft Excel (Microsoft corporations, 2018). Extracted parameters included number of participants, participant demographics, vitals sign/s recorded, comparators, details of the technology used and the findings of the study. The findings of the studies included data on accuracy, failure rates, limitations and important clinical considerations.

### Quality Assessment

Quality assessment was conducted using the QUADAS-2 tool for assessing the quality of diagnostic accuracy studies [6]. This was completed independently by two researchers (MB and DC), and discrepancies were resolved by discussion.

### Data Analysis

Where the research topics were conceptually very broad and where studies addressed noncomparable research questions and vital signs, a narrative review was conducted. Studies describing results using Bland–Altman analysis were included in a meta-analysis using Stata MP17 software (Stata Statistical Software: Release 17. College Station, TX: StataCorp LLC). Where studies did not report Bland–Altman analysis, values of mean bias, and 95% limits of agreement were derived from available data, where possible. Forest plots for mean bias and 95% limits of agreement demonstrate the results of the pooled analysis.

A p value of less than 0.1 was considered to be statistically significant for heterogeneity between studies. I^2^ < 40 was considered low heterogeneity, I^2^ > 40%–75% as intermediate heterogeneity, and I^2^ > 75% as substantial heterogeneity.

## Results

**Figure 1.**
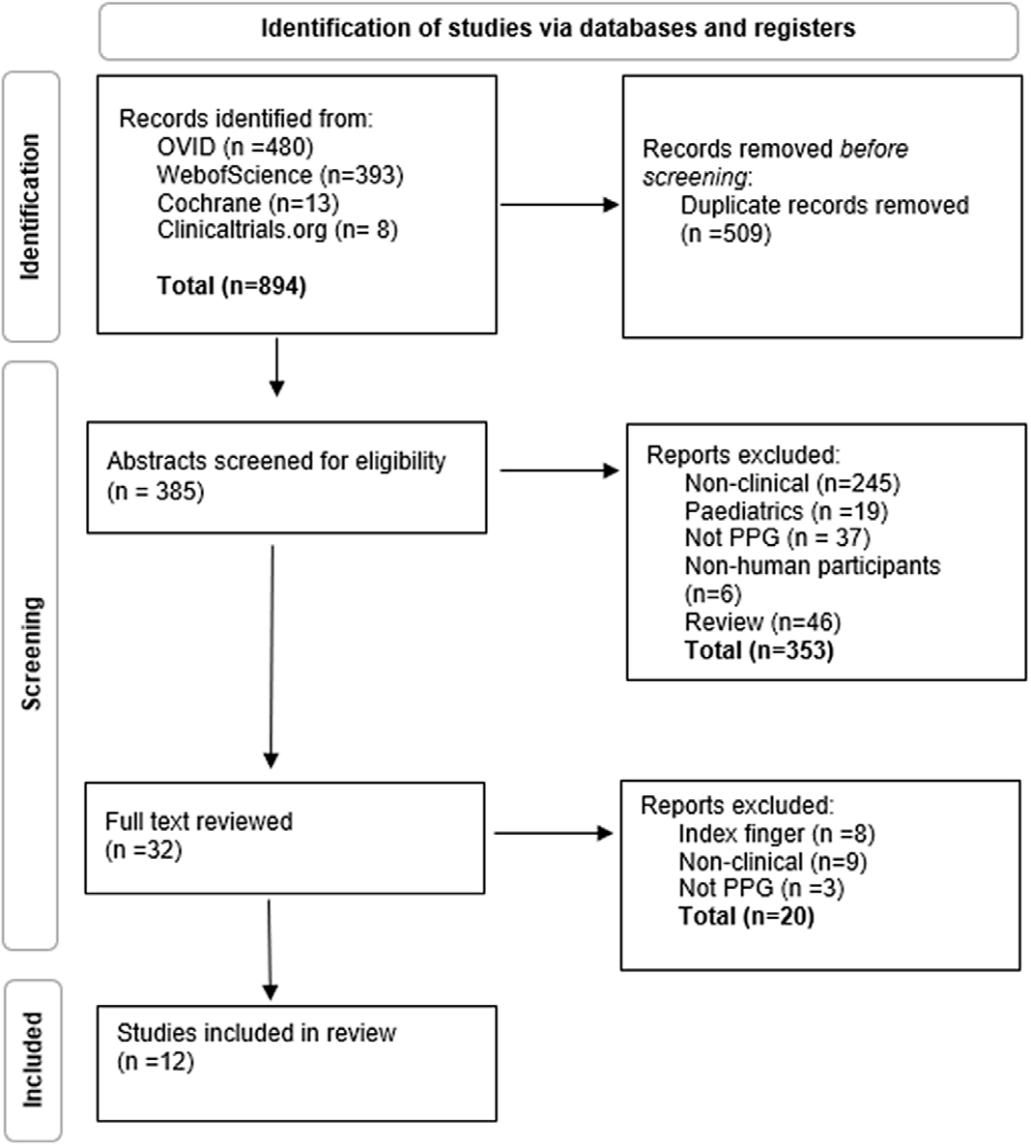
PRISMA flow diagram of the studies included.

Out of the 894 of papers obtained in the initial search, 12 were eligible for inclusion in the review (Fig. 1). These included a total of 654 patients. HR was the most investigated vital sign (*n* = 10) followed by RR (*n* = 2), Sp02 (*n* = 2) and HR variability (*n* = 2) (Table 1). Six studies measured a single physiological parameter and the remainder measured more than one.

**Table 1.** Summary table of the twelve studies included in the systematic review

First Author	Participants (n)	Clinical setting	Vital sign	Camera type	Reference test
Rasche *et al*. 2016 [14]	70	Post cardiac surgery within cardiac ICU	HR	x2 CMOS cameras	ECG
Coppetti *et al*. 2017 [15]	108	Adults requiring monitoring on a chest pain unit	HR	iPhone 4/5	ECG and pulse oximetry
Tarassenko *et al*. 2014 [9]	46	Patients undergoing haemodialysis	HR RR RR Sp02	5 MP Camera	EQ-02 belt (RR), pulse oximetry (HR and Sp02), oscillometric blood pressure monitor
Trumpp *et al*. 2018 [7]	41	Intraoperative monitoring of patients	HR	x4 cameras	N/A
Mamontov *et al*. 2020 [8]	5	Patients undergoing brain surgery	ACP and PAT	CMOS camera	Paired data before and after surgery
Van Gastel 2021 [11]	8	Patients with OSA	HR, RR, Sp02 Desaturation events Desaturation events	X3 Monochrome Cameras	PSG
Unursaikhan *et al*. 2021 [12]	53	26 patients with major depressive disorder	HRV, HR, parasympathetic activation	Web camera	ECG
Yu *et al*. 2021 [16]	20	10 geriatric patients 10 healthy controls	HR HRV HRV	x3 cameras	ECG
Couderc *et al*. 2015 [10]	11	Patients with AF undergoing DC cardioversion	HR	Web camera	ECG
Yan *et al*. 2018 [17]	217	Patients admitted to cardiology wards	HR	iPhone 4S	ECG
Rasche *et al*. 2019 [18]	70	Post cardiac surgery	Optical pulse power of cardiac ejection	CMOS camera	ECG
Hassan *et al*. 2018 [13]	5	Retinal imaging	HR, SP02, RR, and blood vessel diameter	Visuscount 100 fundus camera on a slit lamp	Pulse oximetry

ACP, amplitude of the pulsatile component; AF, atrial fibrillation; CMOS, complimentary metal-oxide semiconductor; DC cardioversion, direct current cardioversion; EQ-02 belt, equivital sensor belt; HR, heart rate; HRV, heart rate variability; MP, mega pixel; OSA, obstructive sleep apnea; PAT, pulse arrival time; PSG, polysomnography; RR, respiratory rate; Sp02, oxygen saturations.

Seven studies measured vital signs within a ward setting; two of these studies were conducted within an intensive care unit, and two studies documented PPG intraoperatively [7,8].

Two studies assessed patients undergoing a particular procedure (cardioversion and hemodialysis) [9,10], and two studies used the technology to aid in the diagnosis of a particular medical condition (obstructive sleep apnea (OSA) and major depressive disorder) [11,12].

Ten studies used a camera to detect skin PPG; one study used retinal blood flow to detect HR, RR, and Sp02 [13]. One further study measured PPG waveform differences of the brain before and after surgery [8].

### Quality Assessment

**Figure 2.**
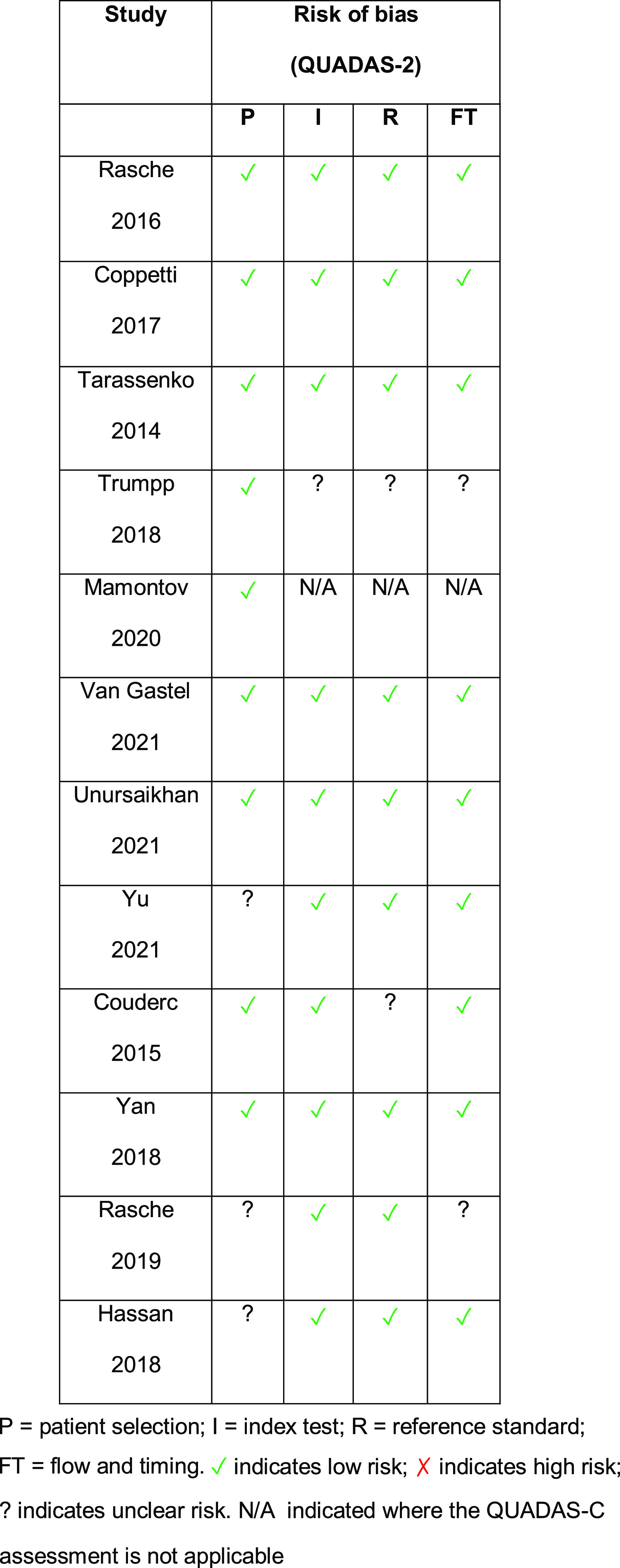
Results using the QUADAS-2 tool for assessment of the quality of each of the selected studies.

Following application of the QUADAS-2 tool for the quality assessment of diagnostic accuracy studies, the majority of the studies demonstrated an overall acceptable quality. The results are displayed below (Fig. 2).

### Heart Rate

HR was the most studied physiological parameter, with a total of ten studies measuring cPPG HR. Six compared cPPG signals to ECG, three compared to pulse oximetry, one compared HR to polysomnography (PSG) data, and one did not have a comparator.

Trumpp *et al.* (2018) was the only study which attempted to measure intra-operative HR [7]. The authors recorded 30 minutes of intra-operative video feed of patients undergoing surgery to the limb or torso. The authors did not report a comparator method or accuracy of the data, however, reported HR was detectable from the acquired data 96% of the time. Van Gastel *et al.* (2021) measured HR and compared this to PSG data for individuals undergoing assessment for OSA [11]. They used three monochrome cameras to capture the full width of the bed of the 8 people being assessed. They demonstrated cPPG was able to detect HR within 2 beats per minute, 92% of the time.

Tarassenko *et al.* (2014) measured cPPG signals from 46 patients undergoing hemodialysis [9]. They used a high-quality 5 M pixel camera positioned approximately 1 m away from the patient. With post data acquisition processing, they used algorithms to measure RR, HR, and Sp02. They demonstrated the HR values obtained from cPPG were very similar to the reference HR obtained from a pulse oximeter/ear lobe probe. They represented their results in a graph showing a 10-minute recording of the cPPG superimposed over the reference index finger pulse oximeter which appears to show very close similarities between the two waveforms. The mean absolute error (MAE) was approximately 3 beats per minute due a period of stillness which is reflective of the MAE of a pulse oximeter.

Coppetti *et al.* (2018) aimed to assess the accuracy of multiple smart phone applications which used contact and contactless PPG methods [15]. They assessed the accuracy of two cPPG downloadable applications, “What’s My Heart Rate” (WMHR) and “Cardiio.” These applications use the front camera of a smart phone to obtain cPPG signals from the face of the user. They compared the applications to pulse oximetry with ECG reference values. The cPPG applications had an inferior performance when compared to contact cPPG methods. Cardiio had an MAE of 8.11 and WMHR had an MAE of 7.08; this was compared to the MAE of the pulse oximeter which was 2.0. The contact methods performed worse than pulse oximetry but were superior to the cPPG methods. Explanations as to why the contact and contactless applications preformed differently could include camera quality, uncontrolled lighting and increased noise.

### Heart Rate and Atrial Fibrillation

Yan *et al.* (2018) and Couderc *et al.* (2015) focused their studies on the detection of atrial fibrillation (AF) using cPPG methods [10,17]. They compared the cPPG results to reference ECG data in patients admitted to a cardiology unit.

Couderc *et al.* (2015) assessed cPPG in 11 AF patients who underwent electrical cardioversion in a single center [10]. They used a Red Green Blue (RGB) web camera placed 1 m above the heads of patient’s whist they were still and supine. They recorded the patient’s face as they underwent cardioversion and compared this information to a reference ECG which was taken simultaneously. They found this technology was associated with a 20% error rate but concluded that cPPG, despite itrs limitations, may be a possible method of contactless cardiovascular monitoring.

Yan *et al*. (2018) studied the Cardiio Rhythm smart phone application that was used in Coppetto *et al.*’s 2018 study. They used this application to assess the rate of AF detection in 217 patients. An iPhone 6s front camera was mounted on a holder approximately 30 cm away from a patient’s face. They recorded three consecutive 20 s recordings, and the data were sent to a secure cloud server where the signal was analyzed for the likelihood of a regular or irregular pulse. They found the Cardiio Rhythm application produced an accuracy of 95.4% with a sensitivity of 94.7% (95% confidence interval 87.1%–97.9%) and specificity of 95.8% (95% confidence interval 91.1% –98.1%) for the detection of AF.

### Heart Rate Meta-analysis

**Figure 3.**
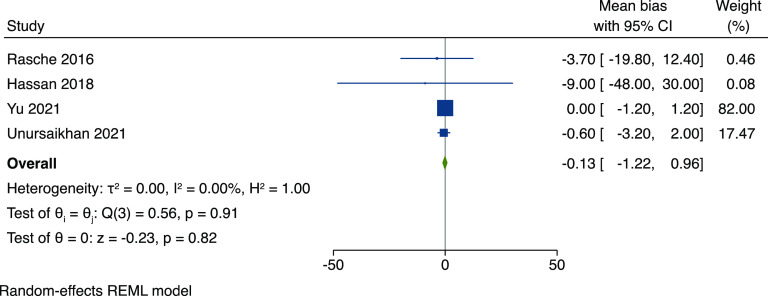
Random effects model forest plot demonstrating the combined heart rate mean difference and 95% confidence intervals, when compared to contact methods of heart rate detection. Overall mean bias is -0.13 (95% CI, -1.22 - 0.96), demonstrated no difference between contact and contactless methods of heart rate monitoring.

Out of the studies measuring HR cPPG, four provided results as mean difference and were considered comparable; these were included in a meta-analysis (Fig. 3). The studies included in the meta-analysis compared cPPG HR to HR obtained from ECG data. This included a total of 148 patients in various clinical settings (Table 2.) The studies reported their results using mean difference or represented this data as a Bland–Altman plot, and the relevant data were extracted.

**Table 2.** Information about the setting, technology, and patients within each study included in the meta-analysis

Author	Patients (n)	Clinical Setting	Technology
Rasche *et al*. 2016 [14]	70	Cardiac ICU	Camera
Hassan *et al*. 2018 [13]	5	Retinal screening	Fundus camera
Yu *et al*. 2021 [16]	20	Geriatric patients	Camera
Unursaikhan *et al*. 2021 [12]	53	MDD screening	Web camera

MDD, major depressive disorder.

Rasche *et al.* (2016) compared cPPG HR to ECG data in 70 patients admitted to a cardiac intensive care unit following elective cardiac surgery [14]. They recorded a mean time of 28 minutes per patients but 5% of their data was discarded due to motion or illumination artefacts. They demonstrated a HR detection of 92.6% per patient and the cPPG data matched their reference (cHR ± 5 bpm) in 83% of the recordings. They demonstrated a mean difference of -3.7 ± 16.1 bpm and measurement error was 87.3%. Both measurement errors decreased as signal to noise (SNR) ratio increased.

Hassan *et al.* (2018) attempted to measure vital signs including HR, RR, and Sp02 using a retinal video feed which detected a PPG waveform from retinal vessels [13]. The authors expressed difficulty when comparing the HR data due to the inability to capture the same time points for contact and retinal data. They found a mean difference of -10 with 95% limits of agreements being -50–30 beats per minute. They found a similar PPG waveform pattern between contact and camera Sp02 and a slightly variable RR around a mean of 20 breaths per minute.

Yu *et al.* (2021) used cPPG to compare HR and HRV between 10 healthy patients and 10 geriatric patients (over 70 years old), admitted to hospital [16]. They used three cameras placed at the end of the patient’s bed at approximately 1.8 m from the subject and compared their cPPG results to reference ECG data. They recorded two sets of recordings: one before and one after a physiotherapy session. They asked the patients to lie supine and remain still for the recordings. The cPPG data provided the most accurate HR values which, when compared to the reference ECG, produced a mean difference of 0.00 bpm with 95% limits of agreement of –1.22–1.29 bpm.

Unursaikhan *et al.* (2021) measured HR, HRV, and parasympathetic activation in a set of healthy volunteers and compared these values to individuals with major depressive disorder (MDD) [12]. The authors developed a remote, contactless screening method for MDD during the COVID-19 pandemic. They recorded several components of sympathetic and parasympathetic nervous activity in participants before, during, and after a mental task, using a webcam device located on a desktop computer. They used inter-beat interval (IBI) and demonstrated a strong correlation (*r* = 0.97, *p* < 0.0001) when compared to reference ECG data.

### Oxygen Saturations and Respiratory Rate

Three studies measured RR and Sp02 using contactless cPPG [9,11,13]. These studies compared the cPPG results to an EQ-02 belt, pulse oximetry, and PSG data. An EQ-02 belt is often strapped to the thorax and includes a belt with an integrated sensor as a contact method of assessing physiological parameters. PSG data are the gold standard method of data during sleep studies, it uses multiple measurements including brain waves, eye movement, oxygen levels, HR, and RR. These parameters were measured in patients undergoing hemodialysis and during assessment for OSA.

Tarassenko *et al.* (2014) measured Sp02 and RR in patients undergoing haemodialysis using an EQ-02 belt and a pulse oximeter compared to a 5-mega pixel camera placed 1 m away from the patient [9]. This study does not report accuracy data, but provides a statement to suggest the camera-based technique is comparable to their reference range. Van Gastel *et al.* (2021) used three monochrome cameras to obtain HR, RR, Sp02, and desaturation events in 8 patients undergoing assessment for OSA [11]. They demonstrated the ability to detect pulse and RR within 2 beats and breaths per minute accuracy 92% and 91% of the time, respectively. They were also able to estimate blood oxygen values within 4 percentage points when compared to the finger oximeter, 89% of the time. Hassan *et al.* (2018) measured cPPG, RR, and Sp02 using a fundus camera to measure PPG signals from the retina. They compared their data to pulse oximetry, but accuracy data were not reported [13].

### Other Physiological Parameters

Other physiological parameters measured using cPPG include amplitude of the pulsatile component (ACP), pulse arrival time (PAT), heart rate variability (HRV), parasympathetic activation (MT), blood vessel diamete, and the optical pulse power of cardiac ejection. These measurements were used in different clinical contexts and patient groups.

Mamontov *et al*. (2020) aimed to measure brain blood flow components and any differences noted before and after brain surgery [8]. They measured ACP and PAT in the exposed cerebral cortex before and after five patients underwent open brain surgery for varying pathologies. The authors used a dark theater room and a green light directed over the cerebrum to elicit the PPG waveform of the surrounding area of brain involved in the surgery. They successfully visualized cerebral cortex blood flow in all patients and in 98% of the open brains. They demonstrated changes in blood flow characteristics as a response to surgery. The ACP decreased within the area resected but increased in surrounding areas, but the PPG waveform shape remained stable; the PAT was increased at the area of resection but decreased in surrounding areas. The authors conclude that contactless PPG could be a cheap and effective way of measuring cerebral blood flow intraoperatively.

Unursaikhan *et al.* (2021) measured HRV and parasympathetic activation for the assessment of MDD [12]. They calculated a Pearson correlation between a single lead ECG and the contactless PPG signal and demonstrated a strong relationship (*r* = 0.97). Their web camera-based non-contact MDD screening tool had a sensitivity of 73% and specificity of 85% but also correlated with the self-rating depression scale scores. The authors thereby conclude contactless PPG can be useful in screening for not only MDD but also high-risk patients.

Yu *et al.* (2021) attempted to measure HRV in a set of geriatric patients using cPPG methods [16]. They were able to estimate HRV in 50% of the data acquired, and these data demonstrated a mean absolute error of 2.4 bpm. The criteria for calculating a meaningful HRV included a data collection time of 2–5 minutes and the heartbeat having originated form the sinoatrial node. This meant exclusion of patients with AF. The data series lacked the appropriate temporal quality.

### Data Acquisition Methods

A wide variety of data acquisition methods were demonstrated in this review. The most common method (*n* = 7) involved more than one camera placed at varying distances from patients (0.5–1.8 m), either above the patient or at the end of the bed [7–9,11,16,18]. There was a wide range of camera resolutions (420 x 320–2592 x 1944 pixels) and number of frames per second (12–100). The number of cameras used ranged from one to a maximum of three. Some studies used a built unit containing the cameras and necessary lighting in attempt to avoid disruption to the clinical setting [7,14]. These camera units remained in place throughout the recording of a patient, and the set up was unique for each study.

Alternative methods of data acquisition included web cameras (*n* = 2), iPhones 4, 5, and 6S (*n* = 2) with the use of a downloadable application (WMHR and Cardiio) [15]. One study used a fundus camera attached to a slit lamp to record PPG from the retina [13].

Most studies used hospital lighting which included ambient lighting and florescent lights within wards and theater environments (*n* = 9). The remaining studies used LED lighting to supplement the luminosity of the environment and one study used green light in a dark environment.

### Limitations Encountered and Influencing Factors

Limitations were usually attributed to patient or environmental factors. Quality of the camera appears to be a factor noticeably responsible for the quality of cPPG signals in studies which compared more than one camera type [15].

Movement artefact is a reason for data loss in almost all studies included in this systematic review. Some studies captured patients while they were sedated or deliberately asked patients to remain still to reduce the effect of motion artefact on their data. An alternative method of reducing motion artefact is the use of prolonged periods of monitoring to capture episodes of stillness which can be used for analysis. In the study conducted by Rasche *et al*. 2016, 5.1% of video recording segments were discarded due to movement and illumination artefact [14]. They also obtained a 17% measurement failure rate but were unable to isolate the causes for these failures. Several other patient factors such as lower blood pressures, use of medications, lower body temperatures, and increasing HRs were found to moderately reduce the performance of cPPG [14,15].

Couderc *et al*. (2015) recruited 11 out of the possible 21 participants available. Four participants were excluded due to the nature of the technology [10]. One participant had a continuous positive airway pressure mask in situ that reduced the performance of cPPG. A participant was wearing make-up during the study, and this obscured the blood volume changes and therefore the PPG signal. Two participants were also excluded due to movement artefact produced by restlessness and inadequate sedation.

Lower light intensity and uncontrolled ambient light source have been attributed to poorer cPPG signal in several studies [14,15]. When cPPG was completed in a theater environment, patient movement, adjustments of the patient’s bed, and cares that obscured the camera were described as difficulties to overcome.

Data on patient demographics such as age, race, and skin color are demonstrated in Table 3. The majority of studies did not report the skin color and ethnicity of the participants therefore drawing conclusions regarding the use of cPPG in different ethnic groups and those with different skin tones are limited.

**Table 3.** Demographics of patients included in each study

First Author	Participants (n)	Ethnicity	Age (range)	Skin colour	Female (%)
Rasche *et al*. 2016 [14]	70	N/A	70 (58–81)	N/A	20 (29%)
Coppetti *et al*. 2017 [15]	108	N/A	65 (52–76)	N/A	35 (32%)
Tarassenko *et al*. 2014 [9]	46	N/A	65 (50–80)	N/A	N/A
Trumpp *et al*. 2018 [7]	41	N/A	65 (53–77)	N/A	17 (41%)
Mamontov *et al*. 2020 [8]	5	N/A	N/A	N/A	N/A
Van Gastel 2021 [11]	8	Caucasian	58 (45–69)	N/A	3 (38%)
Unursaikhan *et al*. 2021 [12]	53	N/A	N/A (18–60)	N/A	22 (41%)
Yu *et al*. 2021 [16]	20	N\A	78 (73–85)	20 white	16 (80%)
Couderc *et al*. 2015 [10]	11	Caucasian	65 (59–71)	N/A	3 (27%)
Yan *et al*. 2018 [17]	217	N/A	70 (56–84)	Von Luschan Skin colour scale: Median 24 (range 6-31)	62 (29%)
Rasche *et al*. 2019 [18]	70	N/A	70 (58–81)	N/A	20 (29%)
Hassan *et al*. 2018 [13]	5	N/A	N/A	N/A	N/A

N/A Nonapplicable due to lack of sufficient data.

## Discussion

Contactless methods of vital signs monitoring have gained academic interest and have demonstrated some promising results in both laboratory and clinical settings. The aim of this review was to determine where cPPG has been tested in a clinical setting and what the findings of these studies were. This was achieved by using a systematic review approach to screen the relevant research articles to determine those where cPPG have been tested in a clinical environment or to assess a particular clinical condition. The review demonstrates several challenges which need to be overcome before consideration of its implementation in clinical practice. One of the main limitations includes the lack of robust research in the form of randomized controlled trials or studies with large populations. Given the majority of the studies are laboratory-based and in healthy volunteers, the current stage of research is focused on assessing the accuracy of cPPG and the adjustments of algorithms in an attempt to provide cPPG results close to the reference values. There is a wide spread of patient populations, methodologies, and conditions studied in the 12 studies included in this systematic review. Although difficult to compare, this review has achieved the aim of demonstrating the clinical contexts where cPPG has been assessed. It also reflects the broad possible application of cPPG and how a relatively cheap and accessible technology can be used in multiple clinical settings for the assessment of several medical conditions.

HR appears to be the most commonly and robustly researched vital sign in all contexts of PPG application. HR has produced the majority of high-quality research, especially in the context of contact methods. There have been several experimental studies and randomized controlled trials measuring HR and cardiac arrhythmias in large populations using other methods of PPG [19,20]. The advantage of contactless HR monitoring in the clinical context includes scenarios where covert observations may be beneficial; for example, in individuals with delirium, dementia, and pediatric populations or where remote monitoring is required, such as remote consultations. As demonstrated by this meta-analysis, cPPG appears to produce relatively accurate results but lacks the quality of evidence when compared to its contact counterparts. This review also demonstrates the variable methods of HR extraction, and the literature demonstrates a move from large bulky cameras to smart phone cameras. With rapidly developing smart phone technology, including the quality of the embedded cameras, there are now several applications available (What’s my HR, Cardiio, FaceBEAT, facereader), which claim to accurately measure HR, but are not yet validated [21,22]. Currently, there are a number of downloadable applications which use index finger PPG data for HR and HRV measurements and these appear to have been more robustly assessed in clinical settings [23,24]. This demonstrates promising results for the improved accessibility and costs of remote HR monitoring.

There has been extensive research demonstrating autonomic dysfunction and its relationship with outcomes in several groups of patients [25,26]. There is a strong correlation between autonomic dysfunction and the risks of complications including cardiorespiratory compromise, which represent over one-third of post-operative complications [27]. HRV as a bedside measurement of autonomic function can be a relatively noninvasive and cheap method of further assessing patients. The European Society of Cardiology and the North American Society of Pacing and Electrophysiology suggest an ECG measurement of around 24 hours to accurately assess the intervals between adjacent QRS complexes [28]. More recent studies have indicated that five-minute recordings produce comparable results [29]. The cPPG method of recording HRV using 5-minute recordings with a smart phone has been explored and demonstrated promising results, when compared to reference ECG data [30]. This supports the use of cPPG from smart devices to calculate HRV in a quick, cheap, and non-invasive manner. A limited number of studies have used this cPPG method of measuring HRV in clinical practice.

Other physiological measurements such as Sp02 have been thoroughly assessed in the controlled settings, and there is a large amount of data available on contact methods for measuring oxygen levels. Here we have only demonstrated two studies that explore the potential of cPPG to measure Sp02 in a clinical context [31]. This is similar for the measurement of blood pressure, where cPPG algorithms have been developed in a laboratory setting but have not yet been tested in a clinical environment [32,33]. Other measurements such as hemoglobin levels [34] and blood glucose levels [35] have been explored in contact PPG methods but not yet evidenced for cPPG methods.

This review was limited to the adult population; however, cPPG has been a topic of interest in the pediatric population owing to the less invasive nature of contactless vital signs monitoring. The value of cPPG has been demonstrated in neonates, particularly pre-term neonates, where contact methods of monitoring can cause distress and skin injury [36]. cPPG has shown promising results in neonates within the neonatal intensive care unit (NICU) [37].

### Limitations

Meta-analyses were conducted where results were deemed comparable. Some studies reported alternative statistical methods or lacked the appropriate details, excluding them form any possible pooled assessment. Out of the ten studies which measured HR with an appropriate comparator, only four were deemed comparable. This highlights the lack of standardization of reporting for these particular research articles. There was also a wide range of data acquisition methods which cannot be accounted for in the meta-analysis.

### Suggestions for Future Research

Given the above limitations, a future direction for cPPG research should include randomized controlled trials or cohort studies with large populations. Further research validating other vital signs (bloods pressure, HRV) in clinical settings should be explored. The development of a protocol for standardizing the reporting of studies can ensure interstudy comparability.

## Conclusion

In this study, we summarized where cPPG has been assessed in real-life clinical settings. Although there is a large body of research on cPPG in the laboratory setting, there remains minimal high-quality studies in the clinical setting. HR remains the most robustly studied vital sign with promise for use in the clinical setting.

## Supplementary material

For supplementary material accompanying this paper visit https://doi.org/10.1017/cts.2023.547. click here to view supplementary material

## References

[1] Allen J. Photoplethysmography and its application in clinical physiological measurement. Physiol Meas. 2007;28(3):R1. doi: 10.1088/0967-3334/28/3/R01. 17322588

[2] Huelsbusch M , Blazek V , Huelsbusch M , et al. Contactless mapping of rhythmical phenomena in tissue perfusion using PPGI. SPIE. 2002;4683:110–117. doi: 10.1117/12.463573.

[3] Hassan MA , Malik AS , Fofi D , et al. Heart rate estimation using facial video: A review. Biomed Signal Process Control. 2017;38:346–360. doi: 10.1016/J.BSPC.2017.07.004.

[4] Pham C , Poorzargar K , Nagappa M , et al. Effectiveness of consumer-grade contactless vital signs monitors: A systematic review and meta-analysis. J Clin Monit Comput. 2022;36(1):41–54. doi: 10.1007/S10877-021-00734-9. 34240262 PMC8266631

[5] Page MJ , McKenzie JE , Bossuyt PM , et al. The PRISMA 2020 statement: An updated guideline for reporting systematic reviews. Int J Surg. 2021;88:105906. doi: 10.1136/BMJ.N71. 33789826

[6] Whiting PF , Rutjes AWS , Westwood ME , et al. QUADAS-2: A revised tool for the quality assessment of diagnostic accuracy studies. Ann Intern Med. 2011;155:529–536. doi: 10.7326/0003-4819-155-8-201110180-00009. 22007046

[7] Trumpp A , Lohr J , Wedekind D , et al. Camera-based photoplethysmography in an intraoperative setting. Biomed Eng Online. 2018;17:1–19. doi: 10.1186/S12938-018-0467-7. PMC5853087 29540189

[8] Mamontov OV , Shcherbinin AV , Romashko RV , et al. Intraoperative imaging of cortical blood flow by camera-based photoplethysmography at green light. Appl Sci. 2020;10:6192. doi: 10.3390/APP10186192.

[9] Tarassenko L , Villarroel M , Guazzi A , et al. Non-contact video-based vital sign monitoring using ambient light and auto-regressive models. Physiol Meas. 2014;35(5):807–831. doi: 10.1088/0967-3334/35/5/807. 24681430

[10] Couderc JP , Kyal S , Mestha LK , et al. Detection of atrial fibrillation using contactless facial video monitoring. Hear Rhythm. 2015;12:195–201. doi: 10.1016/J.HRTHM.2014.08.035. 25179488

[11] Van Gastel M , Stuijk S , Overeem S , et al. Camera-based vital signs monitoring during sleep - a proof of concept study. IEEE J Biomed Heal Informatics. 2021;25:1409–1418. doi: 10.1109/JBHI.2020.3045859. 33338025

[12] Unursaikhan B , Tanaka N , Sun G , et al. Development of a novel web camera-based contact-free major depressive disorder screening system using autonomic nervous responses induced by a mental task and its clinical application. Front Physiol. 2021;12:642986. doi: 10.3389/FPHYS.2021.642986. 34054567 PMC8160373

[13] Hassan H , Jaidka S , Dwyer VM , et al. Assessing blood vessel perfusion and vital signs through retinal imaging photoplethysmography. Biomed Opt Express. 2018;9(5):2351–2364. doi: 10.1364/BOE.9.002351. 29760993 PMC5946794

[14] Rasche S , Trumpp A , Waldow T , et al. Camera-based photoplethysmography in critical care patients. Clin Hemorheol Microcirc. 2016;64(1):77–90. doi: 10.3233/CH-162048. 26890242

[15] Coppetti T , Brauchlin A , Müggler S , et al. Accuracy of smartphone apps for heart rate measurement. Eur J Prev Cardiol. 2017;24(12):1287–1293. doi: 10.1177/2047487317702044. 28464700

[16] Yu X , Laurentius T , Bollheimer C , et al. Noncontact monitoring of heart rate and heart rate variability in geriatric patients using photoplethysmography imaging. IEEE J Biomed Heal Informatics. 2021;25:1781–1792. doi: 10.1109/JBHI.2020.3018394. 32816681

[17] Yan BP , Lai WHS , Chan CKY , et al. Contact-free screening of atrial fibrillation by a smartphone using facial pulsatile photoplethysmographic signals. J Am Heart Assoc. 2018;7(8):e008585. doi: 10.1161/JAHA.118.008585. PMC6015414 29622592

[18] Rasche S , Trumpp A , Schmidt M , et al. Remote photoplethysmographic assessment of the peripheral circulation in critical care patients recovering from cardiac surgery. Int Congr Ser. 2019;52:174–182. doi: 10.1097/SHK.0000000000001249. 30113390

[19] Altini M , Amft O. HRV4Training: large-scale longitudinal training load analysis in unconstrained free-living settings using a smartphone application, Annu Int Conf IEEE Eng Med Biol Soc, 2016:2016, 2610–2613. doi: 10.1109/EMBC.2016.7591265. 28268857

[20] Turakhia MP , Desai M , Hedlin H , et al. Rationale and design of a large-scale, app-based study to identify cardiac arrhythmias using a smartwatch: The apple heart study. Am Heart J. 2019;207:66–75. doi: 10.1016/J.AHJ.2018.09.002. 30392584 PMC8099048

[21] Benedetto S , Caldato C , Greenwood DC , et al. Remote heart rate monitoring - assessment of the facereader rPPg by Noldus. PLoS One. 2019;14(11):e0225592. doi: 10.1371/JOURNAL.PONE.0225592. 31756239 PMC6874325

[22] Kwon S , Kim H , Park KS. Validation of heart rate extraction using video imaging on a built-in camera system of a smartphone, Annu Int Conf IEEE Eng Med Biol Soc, 2012:2012, 2174–2177. doi: 10.1109/EMBC.2012.6346392. 23366353

[23] McManus DD , Lee J , Maitas O , et al. A novel application for the detection of an irregular pulse using an iPhone 4S in patients with atrial fibrillation. Hear Rhythm. 2013;10:315–319. doi: 10.1016/J.HRTHM.2012.12.001. PMC3698570 23220686

[24] Pipitprapat W , Harnchoowong S , Suchonwanit P , et al. The validation of smartphone applications for heart rate measurement. Ann Med. 2018;50(8):721–727. doi: 10.1080/07853890.2018.1531144. 30269602

[25] Hillebrand S , Gast KB , De Mutsert R , et al. Heart rate variability and first cardiovascular event in populations without known cardiovascular disease: Meta-analysis and dose-response meta-regression. Europace. 2013;15(5):742–749. doi: 10.1093/EUROPACE/EUS341. 23370966

[26] Stein PK , Schmieg RE , El-Fouly A , et al. Association between heart rate variability recorded on postoperative day 1 and length of stay in abdominal aortic surgery patients. Crit Care Med. 2001;29(9):1738–1743. doi: 10.1097/00003246-200109000-00014. 11546974

[27] Laitio T , Jalonen J , Kuusela T , et al. The role of heart rate variability in risk stratification for adverse postoperative cardiac events. Anesth Analg. 2007;105(6):1548–1560. doi: 10.1213/01.ANE.0000287654.49358.3A. 18042846

[28] Malik M , John Camm A , Thomas Bigger J , et al. Heart rate variability: Standards of measurement, physiological interpretation, and clinical use. Circulation. 1996;93:1043–1065. doi: 10.1161/01.CIR.93.5.1043/FORMAT/EPUB. 8598068

[29] Schroeder EB , Whitsel EA , Evans GW , et al. Repeatability of heart rate variability measures. J Electrocardiol. 2004;37(3):163–172. doi: 10.1016/j.jelectrocard.2004.04.004. 15286929

[30] Shoushan MM , Alexander Reyes B , Rodriguez AM , et al. Contactless heart rate variability (HRV) estimation using a smartphone during respiratory maneuvers and body movement, Annu Int Conf IEEE Eng Med Biol Soc, 2021:2021, 84–87. doi: 10.1109/EMBC46164.2021.9630167. 34891245

[31] Shao D , Liu C , Tsow F , et al. Noncontact monitoring of blood oxygen saturation using camera and dual-wavelength imaging system. IEEE Trans Biomed Eng. 2016;63(6):1091–1098. doi: 10.1109/TBME.2015.2481896. 26415199

[32] Jeong IC , Finkelstein J. Introducing contactless blood pressure assessment using a high speed video camera. J Med Syst. 2016;40(4):1–10. doi: 10.1007/S10916-016-0439-Z. 26791993

[33] Aziz MH , Hasan MK , Mahmood A , et al. Automated cardiac pulse cycle analysis from photoplethysmogram (PPG) signals generated from fingertip videos captured using a smartphone to measure blood hemoglobin levels. IEEE J Biomed Heal Informatics. 2021;25:1385–1396. doi: 10.1109/JBHI.2021.3068658. 33760745

[34] Hasan MK , Aziz MH , Zarif MII , et al. HeLP ME: Recommendations for non-invasive hemoglobin level prediction in mobile-phone environment. JMIR Mhealth Uhealth. 2021. doi: 10.2196/preprints.16806. PMC8063099 33830065

[35] Sen Gupta S , Kwon TH , Hossain S , et al. Towards non-invasive blood glucose measurement using machine learning: An all-purpose PPG system design. Biomed Signal Process Control. 2021;68:102706. doi: 10.1016/J.BSPC.2021.102706.

[36] Wang D , Xu H , Chen S , et al. Medical adhesive-related skin injuries and associated risk factors in a pediatric intensive care unit. Adv Skin Wound Care. 2019;32:176–182. doi: 10.1097/01.ASW.0000553601.05196.FB. 30845071

[37] Paul M , Karthik S , Joseph J , et al. Non-contact sensing of neonatal pulse rate using camera-based imaging: A clinical feasibility study. Physiol Meas. 2020;41(2):024001. doi: 10.1088/1361-6579/AB755C. 32148333

