# Supplementary material for: Clinical applications of contactless photoplethysmography for monitoring in adults: A systematic review and meta-analysis
Source: J Clin Transl Sci. 2023 May 15;7(1):e129. doi: 10.1017/cts.2023.547 (PMC10260340; doi:10.1017/cts.2023.547)
Supplement: Supplementary file 1 [file S2059866123005472sup001.docx]

**Supplementary material**

Search terms

**((ALL=(contactless)) OR ALL=(camera )) OR ALL=(camera-based)**

**ALL=(PPG ) OR ALL=(Photoplethysmography) OR ALL=(iPPG) OR ALL=(rPPG)**

**(((((((((((ALL=(respiratory rate )) OR ALL=(vital signs)) OR ALL=(heart rate)) OR ALL=(blood pressure)) OR ALL=(BP)) OR ALL=(SPo2)) OR ALL=(So2)) OR ALL=(oxygen levels)) OR ALL=(oxygen saturation)) OR ALL=(breathing rate)) OR ALL=(HR))**
